# Association of workplace infection control and perceived organizational support on worker loneliness during the COVID-19 pandemic

**DOI:** 10.3389/fpubh.2025.1558282

**Published:** 2025-04-10

**Authors:** Midori Funada, Shingo Noguchi, Ryutaro Matsugaki, Kiminori Odagami, Ayako Hino, Seiichiro Tateishi, Mayumi Tsuji, Kazuhiro Yatera, Shinya Matsuda, Yoshihisa Fujino

**Affiliations:** ^1^Center for Stress-Related Disease Control and Prevention, University of Occupational and Environmental Health, Kitakyushu, Fukuoka, Japan; ^2^Department of Respiratory Medicine, University of Occupational and Environmental Health, Kitakyushu, Fukuoka, Japan; ^3^Department of Work Systems and Health, Institute of Industrial Ecological Sciences, University of Occupational and Environmental Health, Kitakyushu, Fukuoka, Japan; ^4^Department of Occupational Health Practice and Management, Institute of Industrial Ecological Sciences, University of Occupational and Environmental Health, Kitakyushu, Fukuoka, Japan; ^5^Department of Mental Health, Institute of Industrial Ecological Sciences, University of Occupational and Environmental Health, Kitakyushu, Fukuoka, Japan; ^6^Disaster Occupational Health Center, Institute of Industrial Ecological Sciences, University of Occupational and Environmental Health, Kitakyushu, Fukuoka, Japan; ^7^Department of Environmental Health, School of Medicine, University of Occupational and Environmental Health, Kitakyushu, Fukuoka, Japan; ^8^Department of Preventive Medicine and Community Health, School of Medicine, University of Occupational and Environmental Health, Kitakyushu, Fukuoka, Japan; ^9^Department of Environmental Epidemiology, Institute of Industrial Ecological Sciences, University of Occupational and Environmental Health, Kitakyushu, Fukuoka, Japan

**Keywords:** COVID-19, Japan, loneliness, occupational health, perceived organizational support

## Abstract

**Objective:**

Research has not fully determined the impact of the infection control practices adopted in workplaces during the COVID-19 pandemic on employees’ feelings of loneliness. This study aimed to clarify the relationship between these control measures and employees’ loneliness during the COVID-19 pandemic, including their relationship with perceived organizational support (POS).

**Methods:**

A prospective cohort study was conducted via an internet-based questionnaire. Of the 27,036 responses, data from 10,562 were analyzed. Workplace infection control practices were divided into four groups based on the number of practices adopted by participants. The odds ratios (ORs) of loneliness associated with each workplace infection control practice were calculated using multilevel logistic regression analysis.

**Results:**

Among the participants, 22.1, 23.6, 22.2, and 32.1% worked at companies that implemented 0–2, 3–5, 6–7, and 8 or more infection control practices, respectively. Loneliness was reported by 6.3% of the participants. After adjustments for sex and age, the OR of the group with the fewest infection control practices was 1.91 (95% CI: 1.54–2.37, *p* < 0.001) compared with the group with the most practices adopted. Adjusting the model for household income, education, occupation, telecommuting frequency, and family cohabitation decreased the OR for the group with the fewest infection control practices adopted to 1.54 (95% CI: 1.20–2.99, *p* = 0.001). After adjustments for either POS, these associations became non-significant.

**Discussion:**

Proactive infection control practices at work were positively associated with workers’ experiences of loneliness. Conversely, loneliness owing to fewer infection control practices was attenuated after adjusting for POS.

## 1 Introduction

The practices implemented in 2020 in response to the COVID-19 pandemic caused considerable changes, curtailing or reducing social activities in people’s everyday lives and work environments. Many countries have implemented strategies to mitigate the risk of the 3 Cs: crowded areas, close-contact environments, and closed spaces. The restriction of social interactions, called lockdowns, was also implemented in some countries. In Japan, the government temporarily enacted a state of emergency and recommended the preventive measures for the 3 Cs (1). For example, individuals were advised to avoid going out for meals, drinks, and shopping, and to work from home by telecommuting and teleworking.

Workplace infection control was a major initiative for COVID-19 infection control during the early months of the pandemic. At work, individuals interact in closed spaces for relatively long periods. This leads to the spread of virus owing to the strong infectivity and airborne transmission routes of COVID-19. In Japan, the following practices were recommended and implemented in many workplaces, in accordance with the Guide to Countermeasures for COVID-19 for the Workplace (2): voluntarily restricting or reducing business travel, visitors, and in-person company meetings; encouraging limitations on social events and dinners; enforcing mandatory mask-wearing during work hours; installing partitions and reconsidering office layouts; recommending daily temperature checks and teleworking; and banning eating at desks.

During the COVID-19 pandemic, the infection control practices implemented in workplaces had diverse impacts on mental health, leading to a deterioration of conditions such as depression, anxiety, and insomnia (3, 4). Additionally, loneliness emerged as a significant social issue (5). Loneliness frequently signifies a sense of societal and community isolation (6) and is linked to not only psychological distress but also depression, anxiety, sleep disorders, and other psychiatric disorders (7–9). Loneliness leads to increased morbidity of psychiatric disorders and risk of mortality, including suicide (10, 11). Maintaining physical distance from others and reducing opportunities for communication among workers negatively affected their feelings of loneliness. Research has shown that the number of individuals feeling lonely rose during the COVID-19 pandemic, compared with the period before COVID-19 (12, 13). The proportion of individuals who reported experiencing loneliness varied, with rates ranging between 20 and 55.2% (6, 10, 14–16) in different countries, and reported as 41.4% (17) in Japan.

However, the influence of workplace infection control practices against COVID-19 on loneliness remains unclear. Generally, these practices may have had a negative impact on workers’ mental health and performance because many of them required physical distancing from others and reduced communication opportunities (18). Meanwhile, the implementation of appropriate infection control practices in the workplace may have been positively perceived by employees as an organizational attitude that represents the employer’s concern for the health and safety of employees, thus increasing employees’ perceived organizational support (POS). POS is defined as the general perception of the extent to which an organization values the contributions of its employees and cares about their well-being (19, 20). It represents two logically distinct aspects of the organization’s positive evaluation of one’s contribution and the organization’s consideration for one’s well-being (21). A negative relationship between POS and loneliness at work has been reported (22). We hypothesized that workers’ loneliness increased as more infection control practices were implemented in the workplace. However, positive infection control practices in the workplace may have also increased workers’ POS and moderated their loneliness and its negative effect on their mental health. As such, we examined the relationship between employees’ loneliness and workplace infection control practices, including the relationship with POS.

## 2 Methods

### 2.1 Subjects

**Figure 1:**
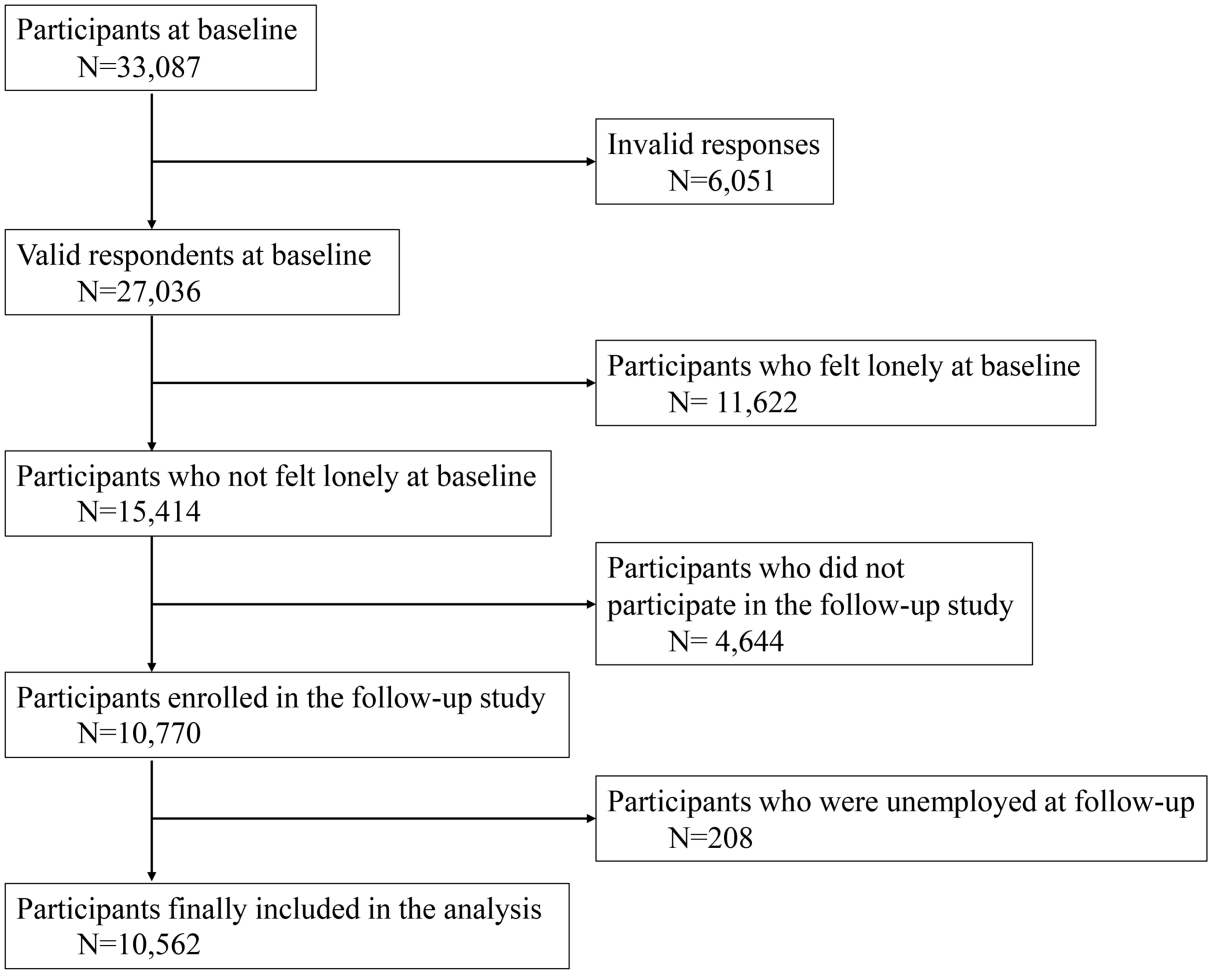
Flowchart of the sample selection procedure in this study. The following exclusion criteria were set: extremely brief response time (<6 min), unusually low body weight (<30 kg) or height (<140 cm), inconsistent answers to similar queries (such as marital status or region of residence), and incorrect answers to questions specifically designed to identify invalid responses.

We conducted a prospective cohort investigation via an online survey. The initial survey was conducted in December 2020, followed by a subsequent survey in December 2021. This research was a component of the the Collaborative Online Research on the Novel-coronavirus and Work (CORoNaWork) project and received approval from the Ethics Committee of the University of Occupational and Environmental Health, Japan (reference number R2-079 and R3-006). All participants in the study were requested to fill out online questionnaires both at the initial phase and during the follow-up. They were informed about the study’s objectives and gave their informed consent. The survey was conducted by Cross Marketing Inc., based in Tokyo, Japan, which has a pool of 4.7 million pre-registered monitors. An initial email was sent to 60,531 men and women aged 20–65 years, and 55,045 responded to the initial screening questions. A total of 33,087 responses fulfilled the inclusion criteria (related to the respondent’s age, sex, region of residence, and employee status), whereas 6,051 were excluded as invalid responses. We set the following exclusion criteria: extremely brief response time (<6 min), unusually low body weight (<30 kg) or height (<140 cm), inconsistent answers to similar queries (such as marital status or region of residence), and incorrect answers to questions specifically designed to identify invalid responses. Among the 27,036 individuals eligible at baseline, 11,622 who felt lonely at baseline were excluded; of the remaining 15,414 eligible for follow-up, 10,770 (69.9%) were enrolled in the follow-up study. Following the exclusion of 208 individuals who were not employed at the time of follow-up, a total of 10,562 individuals were finally included in the analysis. Figure 1 illustrates the selection process. This paper was prepared in accordance with the Strengthening the Reporting of Observational Studies in Epidemiology (STROBE) guidelines (23).

### 2.2 Assessment of workplace infection control practices

We identified 10 specific practices related to workplace infection prevention: limiting or refraining from business trips and visitors; reducing or requesting limits on attendees at social events and dinners; decreasing or limiting in-person internal meetings; enforcing mandatory mask-wearing during work hours; installing partitions and modifying workplace layouts; recommending daily temperature checks at home; promoting telecommuting; forbidding eating at desks; and advising employees to stay home when feeling unwell. The respondents answered “yes” or “no” according to their experiences. We categorized the respondents into four groups based on the number of practices to which they answered “yes”: 0–2, 3–5, 6–7, and 8 or more items.

### 2.3 Assessment of loneliness

We used the University of California, Los Angeles Loneliness Scale (24), and participants were categorized as lonely when they answered “always” to any of following questions: “Do you feel like you do not socialize with others?”; “Do you feel left out from others?”; and “Do you feel isolated from others?”.

### 2.4 Assessment of POS

The assessment of POS was conducted by asking respondents how strongly they agreed with the statement: “Your organization assists its staff in achieving equilibrium between dynamic, efficient work and a lifestyle that promotes health.” This statement was focused on the organizational support for employees’ health and work-life balance. Answers were given on a four-point scale: *strongly agree*, *agree*, *disagree*, and *completely disagree*. The responses were then categorized into four levels of perceived support: *very high* (4 points), *high* (3 points), *low* (2 points), and *very low* (1 point).

### 2.5 Other covariates

Potential confounders included the respondent’s occupation (deskwork, interpersonal, or manual), educational attainment (middle school, high school, or university degree and above), workplace size (<10, 10–49, 50–99, 100–999, or >1,000 employees), household income, living arrangement (with/without family member/s), and number of individuals working at home (at least 4 days per week, at least 2 days per week, at least 1 day per week, at least 1 day per month, and almost never). Participants answered “yes” or “no” to the following inquiry: “Do you feel lonely?”.

### 2.6 Statistical analysis

We calculated the odds ratios (ORs) of loneliness associated with each workplace infection control practice using multilevel logistic regression nested by region of residence. We progressively added subjective ratings of sex, age, education, occupation, household income, family cohabitation, POS, and friendships to the model as adjustment factors. A *p* value less than 0.05 was considered statistically significant. All the data analyses in the study were conducted using Stata17 (Stata, College Station, TX, United States).

## 3 Results

Table 1 lists the fundamental characteristics of the participants, categorized into four groups based on the number of infection control practices adopted in their workplaces. Notably, small companies with fewer than 10 employees accounted for more than half of those that tended to have few infection control measures (0–2). The number of infection control practices in the workplace tended to increase with company size.

**Table 1:** Characteristics of participants according to the number of infection control practices implemented in their workplace.

	Total number of infection control measures in the workplace							
	0–2 (*n* = 2,333)		3–5 (*n* = 2,488)		6–7 (*n* = 2,347)		8 < =(*n* = 3,394)	
	*n*	%	*n*	%	*n*	%	*n*	%
Age in years, mean (SD)	50.3	(9.3)	49.3	(9.7)	49.4	(9.8)	49.5	(9.7)
Sex, men	1,485	(63.7)	1,438	(57.8)	1,387	(59.1)	2081	(61.3)
Job type								
Deskwork	1,063	(45.6)	1,172	(47.1)	1,230	(52.4)	2057	(60.6)
Interpersonal	532	(22.8)	695	(27.9)	640	(27.3)	751	(22.1)
Manual work	738	(31.6)	621	(25.0)	477	(20.3)	586	(17.3)
Education								
Middle school	62	(2.7)	30	(1.2)	16	(0.7)	24	(0.7)
High school	835	(35.8)	673	(27.0)	569	(24.2)	658	(19.4)
University degree and above	1,436	(61.6)	1785	(71.7)	1762	(75.1)	2,712	(79.9)
Enterprise size, number of employees								
<10	1,354	(58.0)	668	(26.8)	315	(13.4)	307	(9.0)
10–49	450	(19.3)	586	(23.6)	387	(16.5)	274	(8.1)
50–99	150	(6.4)	285	(11.5)	237	(10.1)	266	(7.8)
100–999	230	(9.9)	532	(21.4)	744	(31.7)	1,082	(31.9)
1,000<=	149	(6.4)	417	(16.8)	664	(28.3)	1,465	(43.2)
Equivalent income (million JPY)								
40–249	723	(31.0)	495	(19.9)	370	(15.8)	419	(12.3)
250–375	652	(27.9)	694	(27.9)	598	(25.5)	719	(21.2)
376–499	485	(20.8)	602	(24.2)	664	(28.3)	936	(27.6)
≥500	473	(20.3)	697	(28.0)	715	(30.5)	1,320	(38.9)
Family members living together	1934	(82.9)	2084	(83.8)	1979	(84.3)	2,824	(83.2)
Perceived organization support								
Strongly agree	218	(9.3)	193	(7.8)	210	(8.9)	465	(13.7)
Agree	931	(39.9)	1,285	(51.6)	1,366	(58.2)	2,107	(62.1)
Disagree	566	(24.3)	646	(26.0)	562	(23.9)	616	(18.1)
Completely disagree	618	(26.5)	364	(14.6)	209	(8.9)	206	(6.1)
Do you feel lonely?	202	(8.7)	169	(6.8)	134	(5.7)	163	(4.8)
Number of individuals working at home								
At least 4 days per week	409	(17.5)	164	(6.6)	153	(6.5)	451	(13.3)
At least 2 days per week	57	(2.4)	65	(2.6)	107	(4.6)	372	(11.0)
At least 1 day per week	37	(1.6)	43	(1.7)	80	(3.4)	225	(6.6)
At least 1 day per month	15	(0.6)	32	(1.3)	44	(1.9)	130	(3.8)
Almost never	1815	(77.8)	2,184	(87.8)	1963	(83.6)	2,216	(65.3)

Table 2 presents the number and rate of infection control practices in the workplace. “Mandatory mask-wearing during work hours” and “reducing or requesting limits on attendees at social events and dinners” tended to be the most common practices adopted in companies that implemented few and many practices, respectively. Meanwhile, “forbidding eating at desks” was rarely reported.

**Table 2:** Adoption of infection control practices in each respondent grouped by total number of infection control measures implemented.

	Total number of infection control measures in the workplace							
	0–2 (*n* = 2,333)		3–5 (*n* = 2,488)		6–7 (*n* = 2,347)		8 < =(*n* = 3,394)	
	*n*	%	*n*	%	*n*	%	*n*	%
Limiting or refraining from business trips	64	(2.7)	687	(27.6)	1,607	(68.5)	3,314	(97.6)
Limiting or refraining from business visitors	34	(1.5)	365	(14.7)	1,141	(48.6)	3,139	(92.5)
Reducing or requesting limits on attendees at social events and dinners	227	(9.7)	1,631	(65.6)	2,242	(95.5)	3,385	(99.7)
Decreasing or limiting in-person internal meetings	21	(0.9)	605	(24.3)	1723	(73.4)	3,329	(98.1)
Mandatory mask-wearing during work hours	577	(24.7)	2038	(81.9)	2,215	(94.4)	3,354	(98.8)
Installing partitions and modifying workplace layouts	121	(5.2)	1,055	(42.4)	1,658	(70.6)	3,224	(95.0)
Recommending daily temperature checks at home	268	(11.5)	1,390	(55.9)	1746	(74.4)	3,174	(93.5)
Promoting telecommuting	79	(3.4)	269	(10.8)	592	(25.2)	2,226	(65.6)
Forbidding eating at desks	10	(0.4)	123	(4.9)	246	(10.5)	1,290	(38.0)
Advising employees to stay home when feeling unwell	425	(18.2)	1974	(79.3)	2,228	(94.9)	3,370	(99.3)

Values indicate the number and percentage of respondents in each group, categorized by the total number of infection control measures.

Multivariable adjusted ORs for age and sex of individuals experiencing “loneliness” for the number of infection control practices at work are shown in Table 3a. Compared with the group with the highest number of infection control practices at work (8 or more), the ORs for loneliness increased in the groups with fewer infection control practices. The group with the fewest measures (0–2) had significantly higher ORs (1.91, 95% CI: 1.54–2.37, *p* < 0.001) in the age- and sex-adjusted model (Table 3a). When the model was adjusted for household income, occupation, education, and telecommuting frequency (Table 3b), the ORs in the group with the fewest measures (0–2) significantly decreased to 1.56 (95% CI: 1.21–2.01, *p* = 0.001), further decreasing to 1.54 (95% CI: 1.20–2.99, *p* = 0.001) in the model adjusted for family cohabitation (Table 3c). After the model was further adjusted by adding “POS” (Table 3d), the number of workplace infection control practices and feelings of loneliness no longer showed a significant association.

**Table 3:** Association between the number of infection control practices and loneliness.

a. Adjusted for Sex and Age			
Number of infection control initiatives	OR (95%CI)		*p*-value
0-2	1.91	(1.54–2.37)	<0.001
3-5	1.42	(1.14–1.78)	0.002
6-7	1.18	(0.93–1.49)	0.169
8<=	reference		
b. Adjusted for Sex, Age, Household Income, Occupation, Education, and Telecommuting frequency			
Number of infection control initiatives	OR (95%CI)		*p*-value
0-2	1.56	(1.21–2.01)	0.001
3-5	1.34	(1.06–1.71)	0.015
6-7	1.16	(0.91–1.47)	0.233
8<=	reference		
c. Adjusted for Sex, Age, Household Income, Occupation, Education, Telecommuting frequency, and Family Cohabitation			
Number of infection control initiatives	OR (95%CI)		*p*-value
0-2	1.54	(1.20–2.99)	0.001
3-5	1.34	(1.06–1.70)	0.016
6-7	1.16	(0.91–1.48)	0.232
8<=	reference		
d. Adjusted for Sex, Age, Household Income, Occupation, Education, Telecommuting frequency, Family Cohabitation, and POS (Social Resources)			
Number of infection control initiatives	OR (95%CI)		*p*-value
0-2	1.24	(0.96–1.61)	0.103
3-5	1.22	(0.96–1.55)	0.112
6-7	1.12	(0.88–1.42)	0.370
8<=	reference		

## 4 Discussion

We evaluated the relationship between the number of workplace infection control practices and loneliness during the COVID-19 pandemic, and found that fewer infection control practices in the workplace were associated with more workers feeling lonely. This association was attenuated by employees’ POS. Notably, our results contradicted our initial hypothesis. Infection control practices during the COVID-19 pandemic have been reported to be associated with increased loneliness because of the reduced social involvement (14). Many of the workplace infection control practices that we examined in this study focused on avoiding interaction and contact with other people; therefore, we speculated that workers’ loneliness would increase. However, our findings showed that more positive and appropriate infection control practices in the workplace made workers feel less lonely.

We analyzed the underlying reasons for these unexpected associations. First, continuing to work in a facility that had difficulty implementing infection control practices during a rapid increase in COVID-19 cases could have led to an increased fear of infection risk and related events, such as hospital admission and death from COVID-19, which might have led to isolation from society and loneliness. Fear and anxiety related to COVID-19 have been reported to negatively affect people’s mental status (25). However, in line with our current findings, a prior report indicated that the implementation of workplace infection prevention and control practices during the early months of the COVID-19 pandemic and the increase in the number of infection prevention and control practices were positively related to improvements in the work engagement and mental health of employees (26). In addition, a study conducted among community residents reported an association between preventive behaviors against COVID-19 and decreased loneliness (17).

Second, some organizations may not have been interested in infection control even during the peak of the COVID-19 pandemic in 2020. These companies may not only have inadequate management systems for health and safety and for infection control but also little interest in the welfare and well-being of their workers. Working in such establishments may have increased feelings of isolation owing to insufficient support from the company and a lack of solidarity in the workplace. Indeed, a lack of social support leads to deterioration in workers’ mental health (27), while their loneliness decreases with higher perceived social support (28).

Third, our initial hypothesis may have ignored many other possible confounding factors related to infection control and work-related loneliness. The confounders were divided into individual and workplace/company types. Individual-level confounders included differences in individuals’ family relationships, economic status, education, and telecommuting frequency. However, our results revealed that the presence or absence of a family member living with the respondent did not show an association between infection control and loneliness (Tables 3b,c). Thus, feelings of loneliness while living with family members depended on how an individual felt, which might not be reflected in the differences between the presence or absence of family members. Although this study was unable to assess the association between intrafamily relationships and loneliness, our previous study reported that those who spent more time with their families were less likely to feel lonely during the COVID-19 pandemic (29). These relationships may need to be evaluated in the future. Nonetheless, the results regarding the differences between confounding factors and pathways must be interpreted with caution. Conversely, workplace-level confounders included workplace size and type, attitudes toward health and safety initiatives, and health culture. We adjusted for company size and type, but the effects of these factors were small. Further investigation is required to elucidate whether these factors are confounders or underlying mechanisms.

The results show that positive infection control in the workplace is associated with improvement in POS, which reduces loneliness. Before we conducted our study, we believed that workplace infection control could lead to increased risks of loneliness and isolation by reducing opportunities for interaction and communication among workers—our results demonstrated the opposite. POS for infection prevention during the COVID-19 pandemic has been reported to improve work engagement (30). Moreover, given that adjusting for POS lowered the loneliness reported by those working at companies that implemented few infection control practices, we speculated that POS had a positive relationship on workers and contributed to a reduction in loneliness. Alternatively, if workers do not feel supported by the organization because of inadequate implementation of infection control practices, then POS may not be a confounder but rather a mechanism.

Our results indicate that aggressive infection control practices based on precautionary principles have a favorable connection to workers’ feelings of loneliness. Many workplace infection control practices against the spread of COVID-19 were not necessarily evidence-based or empirically implemented based on precautionary principles for public health, and the negative effects of infection control measures, such as isolation, could be greater than the benefits of infection control. Exploring the effectiveness of workplace infection control practices against COVID-19 remains necessary. However, our results emphasized the socio-psychological benefits of the positive implementation of infection control practices in the workplace.

Our study has several limitations. First, workplace infection control was self-reported. However, to our knowledge, no established evaluation method exists other than self-report. We assumed that the estimated chances of misunderstanding by the respondents were low because infection control measures in the workplace were widely described in the guidelines of Japanese workplaces/companies. It is also possible that loneliness might have increased with lower perceived appropriateness, as the adequacy and effectiveness of infection control was not assessed for workers in this study, thus warranting further investigation. Second, the survey on workplace infection control practices was evaluated only at baseline, and it was unclear how the observation timing affected the results of the relationship between infection control measures in the workplace and loneliness. Many companies may have increased the number of their infection control practices as the COVID-19 pandemic lingered, and longer periods of infection control may be considered to have more psychological impact than shorter periods. Notably, these possible misclassifications may have underestimated the original association; therefore, our arguments were not affected by this issue. Third, in this study, the association between the level of infection control measures in the workplace and loneliness was evaluated rather than those between each infection control measure and loneliness. Thus, future studies should examine the latter association. In addition, loneliness may increase with lower perceived appropriateness, as the adequacy and effectiveness of infection control was not assessed for workers in this study. There remains a possibility that unmeasured confounders, such as hobbies, availability of pets, and religion, may have been associated with our results, although we adjusted for several potential confounders. Therefore, further investigation may be needed. Finally, we assessed loneliness using simple self-report items from the University of California, Los Angeles Loneliness Scale. Additionally, POS was also evaluated by a single question; although the measurement validity was untested, the same indicator was previously used (30).

In conclusion, workers experienced less loneliness at workplaces with active infection control practices. This association can be related to human relationships and POS, which can be involved as both confounders or contributing elements in the relationship between the number of infection control practices and loneliness in the workplace.

## Data Availability

The raw data supporting the conclusions of this article will be made available by the authors, without undue reservation.
